# Comparison of the Techniques of Secondary Intraocular Lens Implantation after Penetrating Keratoplasty

**DOI:** 10.1155/2018/3271017

**Published:** 2018-09-12

**Authors:** Katarzyna Krysik, Dariusz Dobrowolski, Ewa Wroblewska-Czajka, Anita Lyssek-Boron, Edward Wylegala

**Affiliations:** ^1^Department of Ophthalmology with Paediatric Unit, St. Barbara Hospital, Trauma Center, Medykow Square 1, 41-200 Sosnowiec, Poland; ^2^Chair and Clinical Department of Ophthalmology, School of Medicine with the Division of Dentistry in Zabrze, Medical University of Silesia in Katowice, Panewnicka 65 St., 40-760 Katowice, Poland; ^3^Department of Ophthalmology, District Railway Hospital, Panewnicka 65 St., 40-760 Katowice, Poland; ^4^Hebei Provincial Eye Hospital, Xingtai, China

## Abstract

**Aim:**

To conduct a retrospective analysis of secondary IOL implantation in patients who underwent PK with no simultaneous IOL implantation.

**Materials and Methods:**

The retrospective study of the secondary implantation of IOLs was conducted in 46 eyes that underwent a primary operation with PK and cataract/lens extraction with no IOL implantation due to capsule rupture or combining corneal or intraocular complications. The minimum period from PK was 12 months. All secondary IOL implantations were performed from January 2011 to August 2017. Aphakic postkeratoplasty patients were treated using one of the surgical techniques for secondary IOL implantation. In-the-bag IOL implantation was possible if the posterior capsule was complete. If the lens capsule remnants were sufficient to provide secure IOL support, an in-the-sulcus IOL implantation was performed. Scleral fixation was offered in eyes with extensive capsular deficiency or the presence of the vitreous body in anterior chamber. BCVA and expected and achieved refraction were evaluated; we included using two biometry devices, and results were compared.

**Results:**

The corrected distance visual acuity (CDVA) before surgery ranged from 0.1 to 0.8 (mean 0.54 ± 0.17). After secondary IOL implantation, CDVA ranged from 0.2 to 0.8 (mean 0.43 ± 0.14) at postoperative 1 month and from 0.3 to 0.9 (mean 0.55 ± 0.15) at postoperative 6 months (*p* < 0.05). Comparison of the final refraction using two methods of biometry showed no statistically significant difference in the group that underwent scleral fixation of the IOL, similar to the findings for the in-the-bag and in-the-sulcus IOL implantation groups. In the scleral-fixation group, *p*=0.55 for the USG biometry technique and *p*=0.22 for the OB technique. *p* values for the IOL-implantation group were *p*=0.49 and *p*=0.44, respectively.

**Conclusion:**

Both implantation methods are safe for the patients. Final refraction is depending on the technique and indication to keratoplasty. Both biometry techniques deliver precise data for IOL choice.

## 1. Introduction

Eyes undergoing penetrating keratoplasty (PK) often have coexisting ocular pathologies which impede simultaneous intraocular lens (IOL) implantation. Lens injury with cataract formation frequently occurs in ocular trauma, intraocular inflammations, and after previous complicated surgeries. A typical triple procedure, consisting of PK, cataract extraction, and IOL implantation, leads to faster visual acuity improvement when compared with staged surgery, and it avoids successive surgeries [1, 2]. Conversely, the large refractive errors associated with triple procedures also need to be taken into account [3]. In some patients—such as those with ocular infections, insufficient capsular bag support, traumatic eyes with moderate to severe posterior segment injury, iris laceration or aniridia, lens subluxation, or loss of zonular integrity—a one-time IOL placement may be difficult and may carry intra- and postoperative risks [4]. Other risk factors associated with the open-sky state and the patient's health status, such as previous ocular surgery, ocular trauma, internal diseases (arterial hypertension, coronary heart disease, diabetes, atherosclerosis, etc.), and the choice of general anaesthesia, may also constitute contraindications for IOL implantation during a single surgery [5–9]. However, secondary IOL implantation is a challenging surgical procedure and demands suitable ocular conditions. Although the methods and timing of the surgical approach have changed over years, the functional reconstruction of the eye is often more difficult than the original attempt at anatomic reconstruction [3, 10]. If there are local or general contraindications for surgery, appropriate contact lens correction can be offered to the patient.

Aphakic patients need successive visual rehabilitation after undergoing PK. In unilateral cases, these patients often present with high ametropia and high anisometropia, which may be very difficult or impossible to correct with other than surgical methods. Implantation of IOLs also helps to separate the anterior from the posterior chamber and to avoid further complications, such as vitreous contact with the endothelium which can lead to corneal decompensation or cystoid macular oedema (CME) [4, 11]. The choice of a secondary implanted IOL includes anterior chamber intraocular lenses (ACIOL), iris-claw or angle-supported lenses, and posterior chamber (retropupillary approach) lenses (PCIOL), such as in-the-bag and in-the-sulcus implanted or scleral- or iris-fixated lenses [3, 12–15]. The main advantage of the PCIOLs is their more physiological location, which is closer to the normal crystalline lens plane, and the greater distance to the cornea [11, 16]. The surgical risks of this method, associated mainly with elderly patients, include a higher frequency of uveal or choroidal bleeding, damage to the blood-aqueous barrier in the ciliary body due to mechanical pressure of the haptics, and endophthalmitis caused by erosion of the scleral sutures and CME [17–19]. This type of surgical approach, although more time-consuming than ACIOL implantation, might have a lower rate of IOL dislocations, glaucoma, or endothelial cell loss with consecutive corneal decompensation and uveitis [18, 20, 21].

The aim of the present study was to conduct a retrospective analysis of secondary IOL implantation in patients who underwent PK with no simultaneous IOL implantation. We report on the surgical technique, its anatomical and refractive outcomes, and complications arising from this kind of treatment.

## 2. Materials and Methods

This was a retrospective study of the secondary implantation of IOLs in 46 eyes that underwent a primary operation with PK and cataract/lens extraction with no IOL implantation. All secondary IOL implantations were performed from January 2011 to August 2017 at the Ophthalmology Department of Saint Barbara Hospital, Trauma Centre, Sosnowiec, Poland. All parts of the data analysis were conducted under the tenets of the Declaration of Helsinki. The most common reason for secondary IOL implantation in our group, rather than simultaneous with keratoplasty, was a lack of the optimal surgical and ocular conditions for safe IOL placement during keratoplasty (Table 1). The minimum period from PK was 12 months. The analysed data from the medical records included demographics, medical history, corrected distance preoperative and postoperative Snellen visual acuity, details and technique of the IOL calculation and IOL calculation formula, and the outcome and complications of surgery. All patients underwent a complete ophthalmic examination, including corrected distance visual acuity, IOP measurement by Goldmann applanation tonometry, and slit-lamp biomicroscopy and fundus examination with a dilated pupil (if examination was possible). In all the eyes, all corneal sutures were removed at least a month prior to IOL power calculation to avoid changes in corneal curvature.

Exclusion criteria were keratoconus or other corneal ectatic disorders, postkeratoplasty ocular diseases (rejection episode, secondary glaucoma), ocular surgery or trauma, and high astigmatism (>6.0 D), which could affect the final refractive treatment.

All PKs and secondary IOL implantations were performed by two experienced surgeons. All patients signed an informed consent form before any surgical procedure. The donor corneas for the PKs originated from our or cooperative tissue banks. We used a Hanna vacuum trephine system (Moria Inc., Antony, France) or Barron radial vacuum trephines (Katena Products Inc. Denville, New Jersey, USA) for trephination.

Aphakic postkeratoplasty patients were treated using one of three surgical techniques for IOL implantation. In-the-bag IOL implantation was possible if the posterior capsule was present (6 cases). If the lens capsule remnants were sufficient to provide secure IOL support, an in-the-sulcus IOL implantation was performed (11 cases). An AcrySof Multipiece MA60AC (Alcon, USA) was implanted in both methods. Indications for scleral fixation were extensive capsular deficiency or the presence of the vitreous body in the anterior chamber with need of an anterior vitrectomy (29 cases). The surgical technique included conjunctival periotomy adjoining the 3 o'clock and 9 o'clock limbus and creation of half-thickness triangular scleral flaps 3 mm posterior to the surgical limbus, avoiding the long posterior ciliary arteries. The corneal incision for lens implantation was made in the superior quadrants of the peripheral cornea, in the 120° area, back from the Vogt's palisades. A transscleral suture passage was made with an ab interno technique, using 10-0 polypropylene suture material, 1.5 mm posterior to the limbus, through the ciliary sulcus. The haptics of the IOL were placed in the ciliary sulcus. The external knot was covered with a scleral flap. A single-piece CZ 70 BD (Alcon, Fort Worth, TX, USA) PMMA IOL with a 7 mm diameter optic was used in all patients. As most IOL power calculations are based on the endocapsular IOL localisation, power adjustment is necessary to account for a more anteriorly positioned lens in the ciliary sulcus.

The intraocular lens power was determined using standard and corneal topography-derived keratometry with the SRK/T formula. We used two independent methods for IOL calculation: ultrasonic biometry (UB) with an A-Scan ultrasonic biometer (Quantel Medical, US) with an applanation technique under topical anaesthesia and interferometry (optical biometry; OB) with an AL-Scan optical biometer (Nidek Co., Ltd., Japan). Keratometric measurements were obtained after removal of the corneal sutures, a minimum of one year after keratoplasty. Primary keratometry and refraction of the operated eyes were unknown.

Because of the potential intrasurgical risks and the duration of this procedure, the scleral fixation was performed under peribulbar or general anaesthesia. Both the in-the-bag and in-the-sulcus IOL implantations were performed under topical anaesthesia. Following surgery, all patients received an intracameral injection of cefuroxime (1 mg).

This retrospective, observational study, according to Polish law, does not require approval by a local bioethics committee.

The XLSTAT-Biomed (Addinsoft SARL, France) computer software was used for statistical analysis and to calculate means and standard deviations. The parameter values were compared using Student's *t*-test or the Mann–Whitney *U* test. For normal and near-normal distributions, a variance analysis was performed using ANOVA, and the homogeneity of variance was then determined using Bartlett's test. The difference between the measurements with different methods was plotted against their mean in a Bland–Altman plot. The 95% limits of agreement (mean difference ± 1.96 standard deviation) gave the distance between the measurements by the methods with 95% confidence. A *p* value of <0.05 was considered statistically significant.

## 3. Results

Between January 2011 and August 2017, 46 secondary IOL implantations were performed in 46 eyes of post-PK patients. The study group consisted of 19 females at the age of 59.95 ± 14.5 (mean ± SD) years (range was: 35–82 years) at the time of the IOL implantation procedure and 27 males at age of 58.19 ± 15.13 (mean ± SD) years (range was: 27–83 years) at the time of the IOL implantation procedure. No statistically significant differences were noted for the group size or age in the female and male groups (*p* < 0.05). There were 46 surgeries, comprising 6 (13%) in-the-bag IOL implantations, 11 (24%) in-the-sulcus IOL implantations, and 29 (63%) scleral fixations of the IOL. All PKs were performed from January 2010 and October 2016. The age at the time of PK was 58.16 ± 14.38 (mean ± SD) (range was: 34–80 years) years for the female group and 56.67 ± 15.37 (mean ± SD) (range was: 25–82 years) years for the male group. The mean interval between PK and secondary IOL implantation was 21 months in the female group and 19 months in the male group. Table 1 shows the surgical techniques used for IOL implantation and the indications for PK.

The indications for PK and cataract/lens extraction were ocular trauma, 28 eyes (61%); inflammation (bacterial origin: 11 eyes, fungal origin: 2 eyes) of the anterior segment of the eye, 13 eyes (28%); and corneal scars and decompensation after ocular surgeries (including refractive intraocular surgery, glaucoma surgery, and pars plana vitrectomy), 5 eyes (11%).

**Figure 1:**
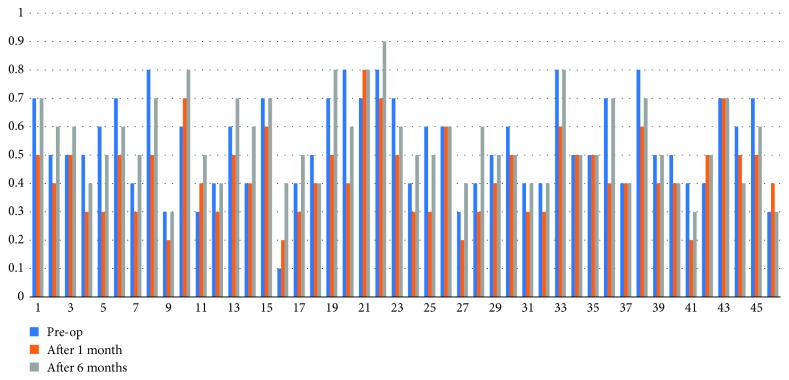
Corrected distance visual acuity at major points before IOL implantation, 1 month after surgery, and 6 months after surgery.

The corrected distance visual acuity (CDVA) before secondary IOL implantation ranged from 0.1 to 0.8 (mean 0.54 ± 0.17). After secondary IOL implantation, the corrected distance visual acuity ranged from 0.2 to 0.8 (mean 0.43 ± 0.14) at postoperative 1 month and from 0.3 to 0.9 (mean 0.55 ± 0.15) at postoperative 6 months (*p* < 0.05).  Figure 1 shows the changes in corrected distance visual acuity from the preoperative period to 1 and 6 months from the secondary IOL implantation.

**Figure 2:**
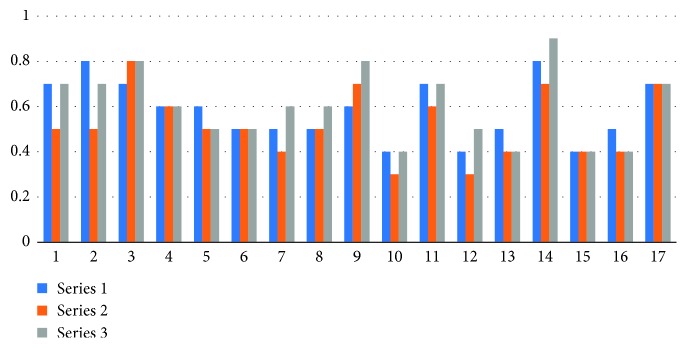
Corrected distance visual acuity. Series 1: before IOL implantation; series 2 : 1 month after surgery; series 3 : 3 months after in-the-bag and in-the-sulcus IOL implantation surgery.

**Figure 3:**
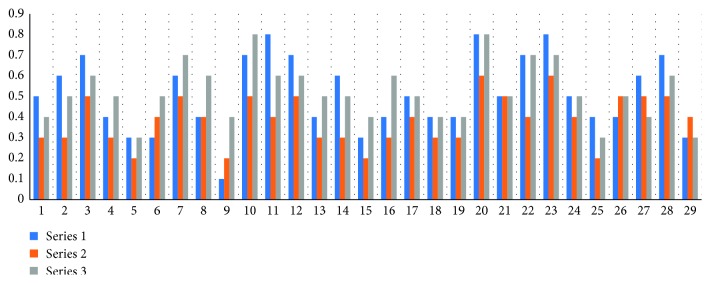
Corrected distance visual acuity. Series 1: before IOL implantation; series 2 : 1 month after surgery; series 3 : 3 months after scleral fixation surgery of the IOL.

Figures 2 and 3 show the corrected distance visual acuity, including the IOL implantation method.

Table 2 shows the corrected distance visual acuity before and after the secondary IOL implantation.

Comparative analysis of the mean CDVA in the three periods of secondary IOL implantation showed statistically significant differences in the values between the preoperative period and 1 month after the secondary IOL implantation (*p*=0.002) and between 1 month and 6 months after the surgery (*p* < 0.001). The preoperative and final mean CDVA in the study group showed no statistically significant difference (*p*=0.69).

**Figure 4:**
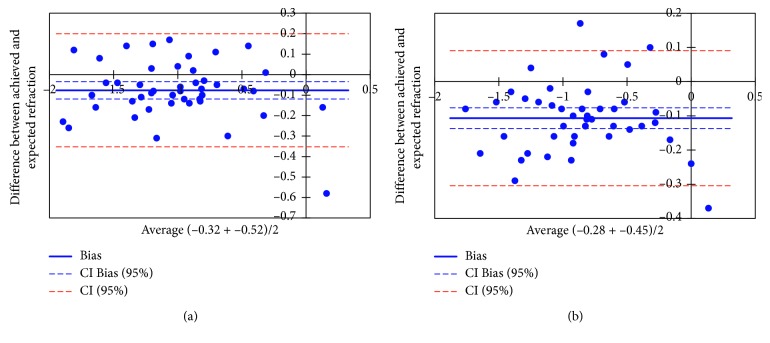
Bland–Altman tests showing the mean difference between the achieved and expected final refraction and the mean refraction in the ultrasound biometry measurement group (a) and the optical biometry measurement group (b).

Figure 4 shows the Bland–Altman plots for the agreement between the two methods of refraction measurement. The dotted lines represent the mean refraction differences between the methods. The interline zones represent the area of 95% limits of agreement.

**Figure 5:**
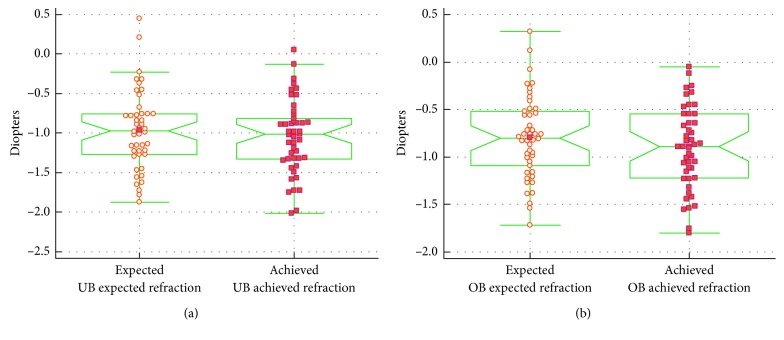
The difference between the expected and achieved refraction for both measurement methods with a myopic shift in the ultrasound biometry measurement group (a) and the optical biometry measurement group (b) (*p* < 0.05).

The differences between the expected and achieved final refraction with a myopic shift and a comparison of the expected and achieved refraction of the whole study group are presented in Figure 5.

**Figure 6:**
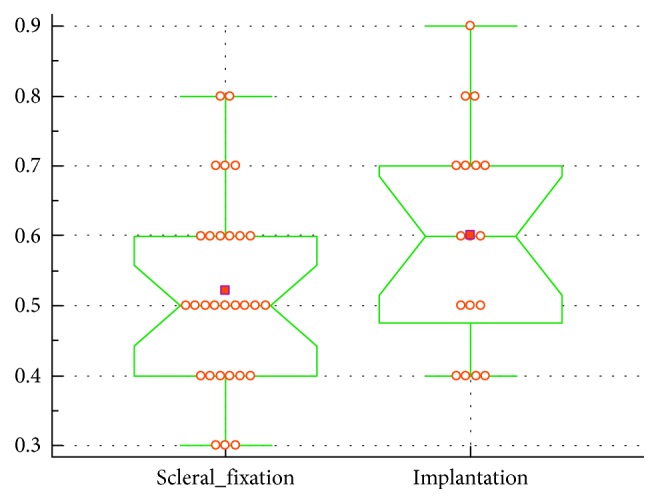
Final corrected distance visual acuity—surgical method comparison (*p*=0.12).

**Figure 7:**
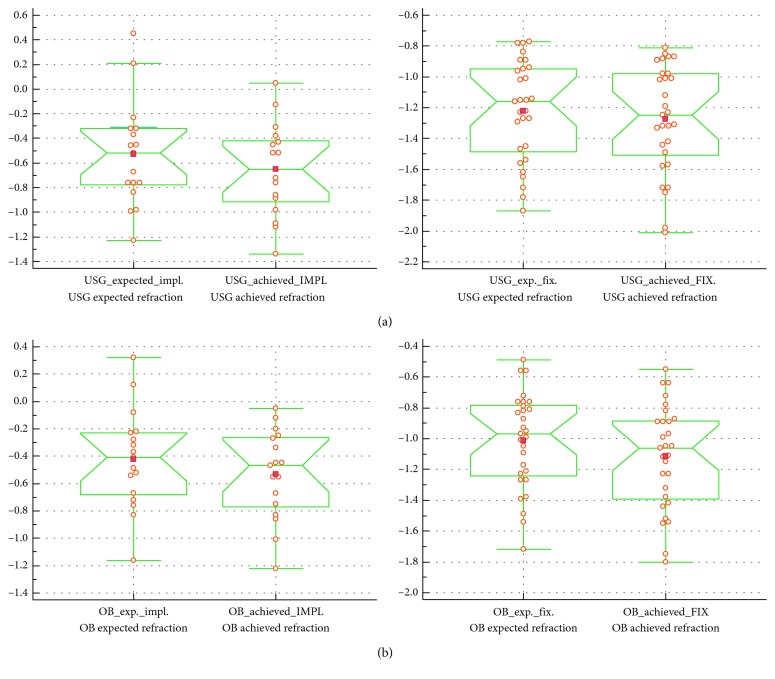
The difference between expected and achieved refraction in both measurement methods with myopic shift in the ultrasound biometry measurement group (a) and the optical biometry measurement group (b) (*p* < 0.05). USG_expected_impl: USB expected refraction; USG_achieved_IMPL: USG achieved refraction; USG_exp._fix.: USG expected refraction; USG_achieved_FIX.: USG achieved refraction; OB_exp._impl.: OB expected refraction; OB_achieved_IMPL: OB achieved refraction; OB_exp._fix.: OB expected refraction; OB_achieved_FIX: OB achieved refraction.

The final corrected distance visual acuity and final refraction in the group of eyes operated with scleral fixation and secondary in-the-bag or in-the-sulcus techniques are shown in Figures 6 and 7.

Comparison of the final refraction using both methods of biometry showed no statistically significant difference in the group that underwent scleral fixation of the IOL, similar to the findings for the in-the-bag and in-the-sulcus IOL implantation groups. In the scleral-fixation group, *p*=0.55 for the USG biometry technique and *p*=0.22 for the OB technique. These values for the IOL-implantation group were *p*=0.49 and *p*=0.44, respectively.

Graft transparency was rated during each follow-up visit. Despite transient partial graft oedema in 6 eyes after scleral fixation (21%), a final full graft transparency was observed at the last control visit in all operated eyes. Other postoperative complications of secondary IOL implantation were glaucoma or ocular hypertension, observed in 7 eyes (24%) after scleral fixation and 2 eyes (18%) after in-the-sulcus IOL implantation. Most of these were treated with 1 or 2 topical agents (timolol 0.5%, brimonidine) with no need for consecutive glaucoma surgery. Moderate, transient irydocyclitis was reported in 4 eyes (14%) after scleral fixation and in 1 eye after in-the-sulcus IOL implantation (9%). Pseudophakic cystoid macular oedema appeared in 2 eyes after scleral fixation (7%), and topical and systemic anti-inflammatory medication was administered. A prolapsed vitreous, updrawn pupil, or anterior synechia was the condition that impeded the easy and safe secondary IOL implantation. In those 2 cases, the patient underwent anterior vitrectomy and anterior synechiolysis. No dislocation of the secondary implanted IOL or endophthalmitis was observed.

## 4. Discussion

The sequential procedure seems to be more accurate for the calculation of the IOL power when compared with the triple procedure (cataract surgery, IOL implantation, and PK). Conversely, the triple procedure allows a faster visual rehabilitation, although it might have a higher risk of intra- and postoperative intraocular infections and other complications [2, 19, 20, 22]. The longer duration of the open-sky state and a higher risk to the patient (including changes in intraocular pressure, posterior capsule rupture, prolapse of the vitreous body, and potential choroidal haemorrhage) frequently forces a need to divide the surgery into two separate approaches [2]. Numerous studies have reported the sequential surgical approach for combined corneal and lens diseases [3, 4, 15, 23].

The indication for secondary IOL implantation is to relieve discomfort or unsatisfactory correction with spectacles or contact lenses for medical, professional, or personal reasons [18]. Precise IOL power calculation is crucial to achieve the expected refraction after lens removal. Many studies have shown less postsurgical refractive error following a two-stage intervention [3, 23]. A very careful surgical approach should be established for globe reconstruction with secondary IOL implantation [10].

The current literature contains insufficient data regarding secondary PCIOL implantation in the eyes after PK and lens removal [3]. The patient profiles differ from those of patients who underwent only lens surgery with no corneal transplantation. The complication profile differed significantly for the patients who underwent scleral fixation versus the other two types of IOL implantation. Our results are compatible with those of Güell et al. [11] who emphasised that the main limitations of the study on secondary IOL implantation were the retrospective approach and the lack of a control group. In addition, a nonhomogeneous group of patients was analysed altogether.

The smaller frequency of intra- and postoperative complications in the groups that underwent the in-the-bag and in-the-sulcus IOL implantation results from an appropriate choice of IOL model, with a large optic and thin long-angulated haptics with more anatomical placement [24, 25]. Postoperative complications of scleral fixation in our study group were comparable to those following secondary scleral fixation in the eyes with no prior corneal surgery [14, 16]. However, we did not measure endothelial cell density, although another aspect of one-stage surgery is a lower risk of endothelial cell loss and stress to the corneal endothelium, due to the open anterior chamber and the lack of a corneal button during IOL implantation. When IOL implantation is performed in the two-stage approach, both the shape and the site of implantation should be taken into account [11, 13, 14, 17, 21, 25].

Ultrasound biometry is still in common use, but optical biometry is now considered the gold standard for IOL calculation. However, our comparison did not reveal any significant differences between these methods. Previous data are not applicable for IOL power evaluation in the eyes after PK. In our sample of patients, the final refraction using ultrasound and optical methods of biometry showed no statistically significantly different values. This demonstrates that even in the scleral-fixation group, despite the large corneal surgical cut, the impact on postoperative refraction is not significant, and the achieved refraction is not statistically significant from that of patients undergoing surgery with small corneal cut. The visual outcomes are consistent with prognostic values: in our sample of patients with secondary IOL implantation, the final mean corrected distance visual acuity was comparable to the preoperative values.

Our findings are compatible with the results of authors who conducted secondary scleral fixation after complicated cataract surgery with no prior corneal surgery [14, 16, 21]. In our group of patients, we did not perform limbal wedge resection or relaxing incisions to improve the second surgery result, as suggested by Geggel [3].

Despite the delayed visual rehabilitation after the sequential surgical approach, the final refractive outcome is crucial [7]. In our opinion, the secondary implantation of the IOL after PK is a very challenging surgery. Because of the complex nature of the indications for surgery and their modalities, the choice of surgical technique and the results of surgical treatment depend on numerous factors. The surgeon has to consider many different factors, including the corneal shape, the anterior chamber depth and its configuration, and the state of the lens capsule and iris. An important consideration for safe lens implantation is accurate timing for the appropriate surgical technique [16, 21].

In summary, our results for the surgical treatment of aphakia after a one-time PK and lens removal show that there is no one-size-fits-all surgical approach. The surgeon's experience and a careful surgical strategy are crucial for the right choice of the IOL lens power and the method of its implantation.

## Figures and Tables

**Table 1:** Surgical techniques used for secondary IOL implantation and the indications for prior keratoplasty with lens extraction.

Characteristics	Total (*n*=46), *N* (%)	Female (*n*=19), *N* (%)	Male (*n*=27), *N* (%)
IOL in-the-bag implantation	6 (13)	2 (33.3)	4 (66.7)
Indication for PK			
Ocular trauma	4 (8.7)	1 (5.26)	3 (11.11)
Ocular inflammation	1 (2.17)	0	1 (3.7)
Previous ocular surgery	1 (2.17)	1 (5.26)	0

IOL in-the-sulcus implantation	11 (24)	7 (63.6)	4 (36.4)
Indication for PK			
Ocular trauma	6 (13.04)	4 (21.06)	2 (7.41)
Ocular inflammation	3 (6.52)	2 (10.52)	1 (3.7)
Previous ocular surgery	2 (4.35)	1 (5.26)	1 (3.7)

IOL scleral fixation	29 (63)	10 (34.5)	19 (65.5)
Indication for PK			
Ocular trauma	18 (39.1)	6 (31.6)	12 (44.45)
Ocular inflammation	9 (19.6)	3 (15.78)	6 (22.23)
Previous ocular surgery	2 (4.35)	1 (5.26)	1 (3.7)

**Table 2:** Corrected distance visual acuity before and after the secondary IOL implantation.

Characteristics	Total *n* (%) (*n*=46)	Preoperative CDVA (range)	Postoperative CDVA (range) After one month	Postoperative CDVA (range) After 6 months
Procedure				
IOL in-the-bag implantation	6	0.5–0.8	0.5–0.8	0.5–0.8
IOL in-the-sulcus implantation	11	0.4–0.8	0.3–0.7	0.4–0.9
IOL scleral fixation	29	0.1–0.8	0.2–0.6	0.3–0.8

Preoperative uncorrected VA was below 0.1, and postoperative UCVA, if compared with corrected results was equal or lower; no more than 2 lines on Snellen charts were observed. Postoperative CDVA was better than or equal to preoperative CDVA in 83.3%, 72.2%, and 68.9% of cases, respectively; 2 cases of in-the-sulcus IOL lost more than 2 lines, and 3 cases of scleral-fixation IOL lost more than 2 lines at the end-point.

## Data Availability

The data describing refractive parameters used to support the findings of this study are included within the article.
